# Supplementary material for: Meta-Analysis and Gene Set Enrichment Relative to ER Status Reveal Elevated Activity of MYC and E2F in the “Basal” Breast Cancer Subgroup
Source: PLoS One. 2009 Mar 9;4(3):e4710. doi: 10.1371/journal.pone.0004710 (PMC2650420; doi:10.1371/journal.pone.0004710)
Supplement: Dataset S1 — GSEA results from first screen. The file contains a zipped archive of the HTML results of the second GSEA screen. Reports for enriched or depleted datasets may be accessed from the “index.html” link within the my_analysis.GseaPreranked.1219975950625.rpt.zip ZIP archive. Enrichment plots are available for each of the 50 most enriched and depleted gene sets. (13.32 MB ZIP) [file pone.0004710.s001.zip › my_analysis.GseaPreranked.1219975950625.rpt.zip!//SERUM_FIBROBLAST_CORE_UP.html]

Details for gene set SERUM\_FIBROBLAST\_CORE\_UP[GSEA]

|  || Dataset | zSco.ERneg.to.ERpos\_collapsed |
| Phenotype | NoPhenotypeAvailable |
| Upregulated in class | na\_pos |
| GeneSet | SERUM\_FIBROBLAST\_CORE\_UP |
| Enrichment Score (ES) | 0.52927095 |
| Normalized Enrichment Score (NES) | 2.3603272 |
| Nominal p-value | 0.0 |
| FDR q-value | 0.0 |
| FWER p-Value | 0.0 |
Table: GSEA Results Summary

  

Fig 1: Enrichment plot: SERUM\_FIBROBLAST\_CORE\_UP      
 Profile of the Running ES Score & Positions of GeneSet Members on the Rank Ordered List

  

| PROBE | GENE SYMBOL | GENE\_TITLE | RANK IN GENE LIST | RANK METRIC SCORE | RUNNING ES | CORE ENRICHMENT || 1 | MSN |  |  | 20 | 6.766 | 0.0160 | Yes |
| 2 | EIF4EBP1 |  |  | 65 | 6.108 | 0.0285 | Yes |
| 3 | UCK2 |  |  | 132 | 5.685 | 0.0382 | Yes |
| 4 | ST3GAL6 |  |  | 155 | 5.534 | 0.0509 | Yes |
| 5 | GGH |  |  | 161 | 5.504 | 0.0648 | Yes |
| 6 | RFC3 |  |  | 164 | 5.493 | 0.0789 | Yes |
| 7 | NUP93 |  |  | 210 | 5.281 | 0.0891 | Yes |
| 8 | RPP40 |  |  | 233 | 5.171 | 0.1009 | Yes |
| 9 | CHEK1 |  |  | 253 | 5.088 | 0.1126 | Yes |
| 10 | WDR77 |  |  | 278 | 4.999 | 0.1238 | Yes |
| 11 | MCM7 |  |  | 289 | 4.970 | 0.1359 | Yes |
| 12 | CRSP8 |  |  | 293 | 4.961 | 0.1485 | Yes |
| 13 | CORO1C |  |  | 299 | 4.934 | 0.1609 | Yes |
| 14 | EXOSC8 |  |  | 306 | 4.923 | 0.1732 | Yes |
| 15 | LMNB2 |  |  | 345 | 4.820 | 0.1828 | Yes |
| 16 | CENPN |  |  | 352 | 4.783 | 0.1948 | Yes |
| 17 | SNRPA |  |  | 368 | 4.749 | 0.2060 | Yes |
| 18 | RANBP1 |  |  | 380 | 4.733 | 0.2174 | Yes |
| 19 | MYBL2 |  |  | 418 | 4.629 | 0.2266 | Yes |
| 20 | SNRPD1 |  |  | 440 | 4.552 | 0.2368 | Yes |
| 21 | C16ORF61 |  |  | 447 | 4.520 | 0.2480 | Yes |
| 22 | NUDT15 |  |  | 456 | 4.493 | 0.2591 | Yes |
| 23 | ENO1 |  |  | 465 | 4.475 | 0.2701 | Yes |
| 24 | TOMM40 |  |  | 561 | 4.216 | 0.2738 | Yes |
| 25 | TUBA1 |  |  | 563 | 4.214 | 0.2846 | Yes |
| 26 | PLAUR |  |  | 604 | 4.138 | 0.2923 | Yes |
| 27 | SMC2 |  |  | 651 | 4.028 | 0.2993 | Yes |
| 28 | DDX11 |  |  | 654 | 4.021 | 0.3095 | Yes |
| 29 | MCM3 |  |  | 656 | 4.019 | 0.3199 | Yes |
| 30 | DCLRE1B |  |  | 696 | 3.939 | 0.3271 | Yes |
| 31 | SNRPB |  |  | 805 | 3.771 | 0.3287 | Yes |
| 32 | C1ORF33 |  |  | 831 | 3.737 | 0.3365 | Yes |
| 33 | PTS |  |  | 884 | 3.648 | 0.3420 | Yes |
| 34 | C18ORF24 |  |  | 894 | 3.635 | 0.3507 | Yes |
| 35 | DCBLD2 |  |  | 895 | 3.635 | 0.3601 | Yes |
| 36 | PPIH |  |  | 943 | 3.562 | 0.3658 | Yes |
| 37 | RNF138 |  |  | 1042 | 3.421 | 0.3672 | Yes |
| 38 | SNRPA1 |  |  | 1064 | 3.399 | 0.3744 | Yes |
| 39 | PSMD14 |  |  | 1069 | 3.392 | 0.3829 | Yes |
| 40 | BRCA2 |  |  | 1126 | 3.331 | 0.3873 | Yes |
| 41 | SMURF2 |  |  | 1129 | 3.330 | 0.3958 | Yes |
| 42 | PFKP |  |  | 1165 | 3.281 | 0.4016 | Yes |
| 43 | CENPJ |  |  | 1172 | 3.270 | 0.4096 | Yes |
| 44 | C12ORF24 |  |  | 1215 | 3.220 | 0.4148 | Yes |
| 45 | EIF4G1 |  |  | 1224 | 3.214 | 0.4225 | Yes |
| 46 | DNAJC9 |  |  | 1243 | 3.198 | 0.4294 | Yes |
| 47 | ITGA6 |  |  | 1288 | 3.149 | 0.4343 | Yes |
| 48 | KIAA0090 |  |  | 1297 | 3.140 | 0.4418 | Yes |
| 49 | UBE2J1 |  |  | 1309 | 3.129 | 0.4491 | Yes |
| 50 | SFRS10 |  |  | 1320 | 3.118 | 0.4564 | Yes |
| 51 | TMEM48 |  |  | 1424 | 3.020 | 0.4564 | Yes |
| 52 | CDCA4 |  |  | 1427 | 3.018 | 0.4641 | Yes |
| 53 | TPM2 |  |  | 1506 | 2.938 | 0.4657 | Yes |
| 54 | RUVBL1 |  |  | 1508 | 2.934 | 0.4733 | Yes |
| 55 | WDHD1 |  |  | 1576 | 2.872 | 0.4756 | Yes |
| 56 | SLC25A5 |  |  | 1599 | 2.850 | 0.4813 | Yes |
| 57 | LOC56902 |  |  | 1605 | 2.845 | 0.4883 | Yes |
| 58 | EPHB1 |  |  | 1675 | 2.780 | 0.4903 | Yes |
| 59 | FLJ10292 |  |  | 1898 | 2.608 | 0.4802 | Yes |
| 60 | SNRPC |  |  | 1939 | 2.581 | 0.4838 | Yes |
| 61 | NUDT1 |  |  | 1996 | 2.533 | 0.4861 | Yes |
| 62 | LRRC40 |  |  | 2008 | 2.524 | 0.4918 | Yes |
| 63 | COQ2 |  |  | 2048 | 2.485 | 0.4953 | Yes |
| 64 | RPN1 |  |  | 2053 | 2.479 | 0.5014 | Yes |
| 65 | H2AFZ |  |  | 2105 | 2.447 | 0.5039 | Yes |
| 66 | MET |  |  | 2188 | 2.394 | 0.5039 | Yes |
| 67 | MT1F |  |  | 2350 | 2.285 | 0.4975 | Yes |
| 68 | STK17A |  |  | 2359 | 2.282 | 0.5029 | Yes |
| 69 | CCT5 |  |  | 2398 | 2.262 | 0.5058 | Yes |
| 70 | WSB2 |  |  | 2459 | 2.219 | 0.5070 | Yes |
| 71 | NDRG1 |  |  | 2463 | 2.218 | 0.5125 | Yes |
| 72 | EBNA1BP2 |  |  | 2478 | 2.211 | 0.5172 | Yes |
| 73 | TXNL2 |  |  | 2484 | 2.208 | 0.5226 | Yes |
| 74 | HMGB1 |  |  | 2520 | 2.191 | 0.5256 | Yes |
| 75 | ACTL6A |  |  | 2632 | 2.128 | 0.5226 | Yes |
| 76 | CDK2 |  |  | 2688 | 2.099 | 0.5239 | Yes |
| 77 | PSMD2 |  |  | 2759 | 2.063 | 0.5239 | Yes |
| 78 | HSPC111 |  |  | 2760 | 2.062 | 0.5293 | Yes |
| 79 | CCND3 |  |  | 2854 | 2.011 | 0.5274 | No |
| 80 | PFN1 |  |  | 2943 | 1.961 | 0.5258 | No |
| 81 | UMPS |  |  | 3124 | 1.877 | 0.5170 | No |
| 82 | RNASEH2A |  |  | 3126 | 1.876 | 0.5218 | No |
| 83 | IL7R |  |  | 3145 | 1.868 | 0.5252 | No |
| 84 | MTHFD1 |  |  | 3258 | 1.814 | 0.5214 | No |
| 85 | RBMX |  |  | 3634 | 1.647 | 0.4972 | No |
| 86 | BRIP1 |  |  | 3774 | 1.596 | 0.4907 | No |
| 87 | PDIA4 |  |  | 3785 | 1.592 | 0.4941 | No |
| 88 | C11ORF24 |  |  | 3831 | 1.578 | 0.4948 | No |
| 89 | FLNC |  |  | 3906 | 1.550 | 0.4932 | No |
| 90 | UAP1 |  |  | 3959 | 1.529 | 0.4932 | No |
| 91 | POLE2 |  |  | 3975 | 1.520 | 0.4960 | No |
| 92 | NCLN |  |  | 4011 | 1.508 | 0.4972 | No |
| 93 | TPRKB |  |  | 4092 | 1.476 | 0.4950 | No |
| 94 | HNRPAB |  |  | 4113 | 1.468 | 0.4972 | No |
| 95 | TNFRSF12A |  |  | 4486 | 1.323 | 0.4724 | No |
| 96 | MAP3K8 |  |  | 4547 | 1.304 | 0.4712 | No |
| 97 | MRPL12 |  |  | 4601 | 1.286 | 0.4705 | No |
| 98 | C1ORF41 |  |  | 4672 | 1.262 | 0.4684 | No |
| 99 | PLOD2 |  |  | 4888 | 1.192 | 0.4552 | No |
| 100 | PSMA7 |  |  | 4890 | 1.191 | 0.4582 | No |
| 101 | HNRPR |  |  | 4913 | 1.185 | 0.4596 | No |
| 102 | DLEU1 |  |  | 4922 | 1.183 | 0.4620 | No |
| 103 | ACTC1 |  |  | 5003 | 1.155 | 0.4590 | No |
| 104 | PTPLB |  |  | 5075 | 1.125 | 0.4565 | No |
| 105 | FARSLA |  |  | 5162 | 1.094 | 0.4528 | No |
| 106 | HAS2 |  |  | 5204 | 1.079 | 0.4524 | No |
| 107 | F3 |  |  | 5288 | 1.053 | 0.4489 | No |
| 108 | TIMM50 |  |  | 5551 | 0.958 | 0.4314 | No |
| 109 | MAPRE1 |  |  | 5601 | 0.944 | 0.4301 | No |
| 110 | POLE3 |  |  | 5825 | 0.870 | 0.4154 | No |
| 111 | NPTN |  |  | 5846 | 0.861 | 0.4161 | No |
| 112 | PSMD12 |  |  | 5876 | 0.852 | 0.4161 | No |
| 113 | PCSK7 |  |  | 5893 | 0.849 | 0.4171 | No |
| 114 | MLF1IP |  |  | 5991 | 0.815 | 0.4119 | No |
| 115 | MCTS1 |  |  | 6129 | 0.775 | 0.4034 | No |
| 116 | GPLD1 |  |  | 6249 | 0.730 | 0.3963 | No |
| 117 | LYPLA2 |  |  | 6621 | 0.615 | 0.3697 | No |
| 118 | SSR3 |  |  | 6643 | 0.609 | 0.3696 | No |
| 119 | SFRS2 |  |  | 6677 | 0.597 | 0.3687 | No |
| 120 | PSMC3 |  |  | 6733 | 0.577 | 0.3660 | No |
| 121 | BCCIP |  |  | 6816 | 0.546 | 0.3612 | No |
| 122 | DUT |  |  | 6839 | 0.539 | 0.3609 | No |
| 123 | IFRD2 |  |  | 6869 | 0.528 | 0.3601 | No |
| 124 | LPAL2 |  |  | 6875 | 0.526 | 0.3610 | No |
| 125 | PDLIM7 |  |  | 6896 | 0.521 | 0.3609 | No |
| 126 | SMS |  |  | 6922 | 0.512 | 0.3603 | No |
| 127 | SPFH1 |  |  | 6942 | 0.505 | 0.3602 | No |
| 128 | C13ORF1 |  |  | 6963 | 0.501 | 0.3599 | No |
| 129 | PITPNC1 |  |  | 7283 | 0.390 | 0.3367 | No |
| 130 | LSM3 |  |  | 7611 | 0.266 | 0.3125 | No |
| 131 | PNN |  |  | 7636 | 0.256 | 0.3113 | No |
| 132 | TDP1 |  |  | 7685 | 0.236 | 0.3083 | No |
| 133 | DLEU2 |  |  | 7765 | 0.204 | 0.3028 | No |
| 134 | NRIP3 |  |  | 7890 | 0.151 | 0.2938 | No |
| 135 | NUP107 |  |  | 8113 | 0.067 | 0.2771 | No |
| 136 | CLCF1 |  |  | 8204 | 0.033 | 0.2703 | No |
| 137 | MT3 |  |  | 8224 | 0.025 | 0.2689 | No |
| 138 | KRR1 |  |  | 8451 | -0.057 | 0.2519 | No |
| 139 | DBNDD1 |  |  | 8471 | -0.066 | 0.2506 | No |
| 140 | RAB3B |  |  | 8594 | -0.126 | 0.2417 | No |
| 141 | UCRC |  |  | 8653 | -0.153 | 0.2376 | No |
| 142 | DYNLT1 |  |  | 8724 | -0.180 | 0.2328 | No |
| 143 | SLC25A40 |  |  | 8815 | -0.216 | 0.2265 | No |
| 144 | VIL2 |  |  | 8844 | -0.228 | 0.2250 | No |
| 145 | NUPL1 |  |  | 8850 | -0.231 | 0.2252 | No |
| 146 | PAX9 |  |  | 8864 | -0.239 | 0.2248 | No |
| 147 | COPB2 |  |  | 8889 | -0.254 | 0.2236 | No |
| 148 | DCK |  |  | 8935 | -0.280 | 0.2209 | No |
| 149 | ID3 |  |  | 8993 | -0.308 | 0.2174 | No |
| 150 | ID2 |  |  | 9049 | -0.335 | 0.2141 | No |
| 151 | MKKS |  |  | 9066 | -0.345 | 0.2138 | No |
| 152 | JTV1 |  |  | 9145 | -0.379 | 0.2088 | No |
| 153 | STX3 |  |  | 9162 | -0.388 | 0.2086 | No |
| 154 | NOLA2 |  |  | 9385 | -0.499 | 0.1930 | No |
| 155 | SDC1 |  |  | 9416 | -0.521 | 0.1921 | No |
| 156 | CBX1 |  |  | 9417 | -0.522 | 0.1934 | No |
| 157 | RGS13 |  |  | 9599 | -0.616 | 0.1813 | No |
| 158 | GSTCD |  |  | 9819 | -0.736 | 0.1665 | No |
| 159 | RUVBL2 |  |  | 10006 | -0.848 | 0.1546 | No |
| 160 | SNRPE |  |  | 10109 | -0.910 | 0.1492 | No |
| 161 | NUP85 |  |  | 10482 | -1.147 | 0.1239 | No |
| 162 | HNRPA3 |  |  | 10647 | -1.279 | 0.1147 | No |
| 163 | TFPI2 |  |  | 10754 | -1.356 | 0.1101 | No |
| 164 | C16ORF34 |  |  | 10823 | -1.408 | 0.1086 | No |
| 165 | HMGN2 |  |  | 10916 | -1.492 | 0.1055 | No |
| 166 | MRPS28 |  |  | 10958 | -1.537 | 0.1064 | No |
| 167 | GNG11 |  |  | 11366 | -1.898 | 0.0803 | No |
| 168 | RNF41 |  |  | 11501 | -2.037 | 0.0754 | No |
| 169 | MRPS16 |  |  | 11541 | -2.078 | 0.0778 | No |
| 170 | SAR1B |  |  | 12598 | -3.430 | 0.0064 | No |
| 171 | IPO4 |  |  | 12777 | -3.812 | 0.0027 | No |
| 172 | MNAT1 |  |  | 12891 | -4.114 | 0.0048 | No |
| 173 | POLR3K |  |  | 12995 | -4.441 | 0.0085 | No |
| 174 | EMP2 |  |  | 13273 | -6.228 | 0.0036 | No |
Table: GSEA details [plain text format]

  

Fig 2: SERUM\_FIBROBLAST\_CORE\_UP: Random ES distribution      
 Gene set null distribution of ES for **SERUM\_FIBROBLAST\_CORE\_UP**
